# Prevalence and drivers of malaria infection among asymptomatic and symptomatic community members in five regions with varying transmission intensity in mainland Tanzania

**DOI:** 10.1186/s13071-024-06639-1

**Published:** 2025-01-24

**Authors:** Gervas A. Chacha, Filbert Francis, Salehe S. Mandai, Misago D. Seth, Rashid A. Madebe, Daniel P. Challe, Daniel A. Petro, Dativa Pereus, Ramadhani Moshi, Rule Budodo, Angelina J. Kisambale, Ruth B. Mbwambo, Catherine Bakari, Sijenunu Aaron, Daniel Mbwambo, Stella Kajange, Samuel Lazaro, Ntuli Kapologwe, Celine I. Mandara, Deus S. Ishengoma

**Affiliations:** 1 National Institute for Medical Research, Dar es Salaam, Tanzania; 2 National Institute for Medical Research, Tanga Research Centre, Tanga, Tanzania; 3 University of Dar Es Salaam, Dar es Salaam, Tanzania; 4 Muhimbili University of Health and Allied Sciences, Dar es Salaam, Tanzania; 5 National Malaria Control Programme, Dodoma, Tanzania; 6 President’s Office, Regional Administration and Local Government, Dodoma, Tanzania; 7 Directorate of Preventive Services, Ministry of Health, Dodoma, Tanzania; 8 Department of Biochemistry, Kampala International University in Tanzania, Dar es Salaam, Tanzania

**Keywords:** Malaria, Symptomatic infections, Asymptomatic infections, Drivers of malaria infections, *Plasmodium falciparum*, Tanzania

## Abstract

**Background:**

Despite implementation of effective interventions in the past two decades, malaria is still a major public health problem in Tanzania. This study assessed the prevalence and drivers of malaria infections among symptomatic and asymptomatic members of selected communities from five regions with varying endemicity in mainland Tanzania.

**Methods:**

A cross-sectional community survey was conducted in five districts, including one district/region in Kagera, Kigoma, Njombe, Ruvuma and Tanga from July to August 2023. Participants aged ≥ 6 months were recruited and tested using rapid diagnostic tests (RDTs). Demographic, anthropometric, clinical, parasitological, type of house, and socio-economic status (SES) data were captured using structured questionnaires. Associations between parasite prevalence and potential drivers were determined by logistic regression, and the results were presented as crude (cOR) and adjusted odds ratios (aOR), with 95% confidence intervals (CIs).

**Results:**

Among 10,228 individuals tested, 3515 (34.4%) had positive results by RDTs. The prevalence of malaria varied from 21.6% in Tanga to 44.4% in Kagera, and from 14.4% to 68.5% among the different villages (*P* < 0.001). The odds of malaria infections were higher in males (aOR = 1.32, 95% CI 1.19–1.48, *P* < 0.001), under-fives (aOR = 2.02, 95% CI 1.74–2.40, *P* < 0.001), schoolchildren [aged 5–9 years (aOR = 3.23, 95% CI 1.19–1.48, *P* < 0.001) and 10–14 years (aOR = 3.53, 95% CI 3.03–4.11, *P* < 0.001)], and non-bednet users (aOR = 1.49, 95% CI 1.29–1.72, *P* < 0.001). Individuals from households with low SES (aOR = 1.40, 95% CI 1.16–1.69, *P* < 0.001), or living in houses with open windows (aOR = 1.24, 95% CI 1.06–1.45, *P* < 0.001) and/or holes on the walls (aOR = 1.43, 95% CI 1.14–1.81, *P* < 0.001) also had higher odds.

**Conclusions:**

Malaria prevalence varied widely across regions and villages, and the odds of infections were higher in males, schoolchildren, non-bednet users, and individuals with low SES or living in houses with open windows and/or holes on the walls. The identified vulnerable groups and hotspots should be targeted with specific interventions to reduce the disease burden and support the ongoing malaria elimination efforts in Tanzania.

**Graphical Abstract:**

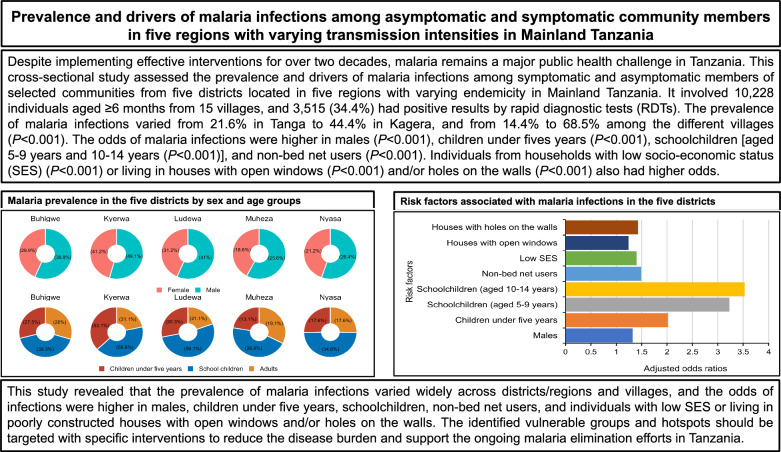

**Supplementary Information:**

The online version contains supplementary material available at 10.1186/s13071-024-06639-1.

## Background

Tanzania has experienced a significant decline in malaria burden over the past two decades [1], and this has been mainly attributed to deployment and implementation of effective interventions recommended by the World Health Organization (WHO) for enhanced control and progressing to malaria elimination [2, 3]. Despite these significant changes in malaria epidemiology, malaria is still a leading public health problem in Tanzania, and the country experiences heterogeneous transmission at macro- and micro-geographical levels [2, 4]. According to the WHO World malaria report 2023, there were an estimated 249 million malaria cases and 608,000 deaths globally in 2022, and the majority of these were from the WHO African Region (WHO-AFRO) [5]. Tanzania was among 11 countries with the highest malaria burden, accounting for over 4.4% of all malaria deaths globally in that year [5]. Currently, Tanzania experiences varying malaria burden, with some areas having very high and stable transmission intensity, while others have very low transmission intensity [6, 7]. Over 93.0% of the Tanzanian population lives in areas where transmission occurs, and risk of malaria infection [8]. *Plasmodium falciparum* is the leading cause of malaria, accounting for 96.0% of cases, and the rest (4.0%) are due to other species including *Plasmodium ovale*, *Plasmodium malariae*, and *Plasmodium vivax* [9, 10].

Despite the progress made, Tanzania still faces challenges that may limit its progress toward malaria elimination. In recent years, resistance to insecticides by mosquito vectors [11–13] and to widely used antimalarial drugs by parasites [14, 15] have been reported, including the emergence of artemisinin partial resistance (ART-R) in Kagera region [16, 17]. In addition, parasites with histidine-rich protein 2/3 (*hrp2/3*) gene deletions that affect the performance of histidine-rich protein 2 (HRP2)-based rapid diagnostic tests (RDTs) have been reported but at low prevalence [18, 19]. There is also a high risk of emergence and spread of an invasive *Anopheles stephensi* vector that has been reported in Kenya and other countries in the Horn of Africa, particularly in Ethiopia [20–22]. Thus, the country needs to intensify surveillance to detect the emergence of new threats, track and control the already reported threats, and ensure that progress to the elimination targets is not impacted.

The Tanzania National Malaria Control Programme (NMCP) has been implementing most of the key effective interventions as recommended by WHO for vector control and case management together with other initiatives such as preventive therapies. For vector control, NMCP in Tanzania uses insecticide-treated nets (ITNs), indoor residual spraying (IRS), and larval source management (LSM) using effective biolarvicides [2]. Case management interventions focus primarily on timely and accurate parasitological diagnosis and confirmation with RDTs and effective treatment using artemisinin-based combination therapy (ACT) [3, 8]. Although WHO recommends several preventive therapies [23], Tanzania is currently employing intermittent preventive treatment for pregnant women (IPTp) using sulfadoxine-pyrimethamine (SP) only, targeting the delivery of two or more doses to all pregnant women from their first trimester [24]. Plans are also underway to deploy and implement intermittent preventive treatment for schoolchildren and infants (IPTi; also known as perennial malaria chemoprevention). Thus, strategies are urgently needed to support the ongoing control efforts and enhance progress towards the 2030 malaria elimination targets.

Due to scaled-up interventions in Tanzania, the malaria burden has declined significantly, with the overall prevalence dropping from 18.0% in 2008 to 8.0% in 2022 [1]. The transmission of malaria is currently heterogeneous, with a large proportion of the regions (about 38.0%) located in areas with low to very low transmission intensity (covering the central corridor, northern, and south-western parts), sandwiched between moderate-transmission areas on both sides and high malaria burden in the north-western, north-eastern, southern, and western regions [2, 4, 25]. Hence, novel interventions are urgently needed to support the NMCP’s efforts to get back on track and progress towards the national elimination targets through deployment and use of new and current interventions based on the local burden and transmission intensity [3].

In Tanzania, extensive research, surveillance and control efforts have been undertaken over the past two decades [25], and have contributed to increased awareness, improved diagnostics, and more effective treatment strategies for malaria [26]. However, amidst these commendable efforts, relatively little attention has been given to asymptomatic individuals, leaving a significant gap in our understanding of their prevalence and contribution to malaria transmission in the communities. Asymptomatic individuals serve as reservoirs of infection and contribute to the persistence of transmission, impeding malaria reduction and elimination efforts [27]. The scarcity of data on the scope and prevalence of asymptomatic malaria presents a critical gap in Tanzania’s malaria control efforts. To address the gap, this study aimed to assess the prevalence and drivers of malaria infection among community members (including asymptomatic and symptomatic individuals) through a community cross-sectional survey (CSS) in five regions in mainland Tanzania with varying transmission intensity. The findings from this study will aid the NMCP and other stakeholders in enhancing the current surveillance system and closing the gaps in the ongoing elimination efforts by targeting areas with high prevalence of malaria infection and vulnerable groups.

## Methods

### Study design and sites

**Fig. 1:**
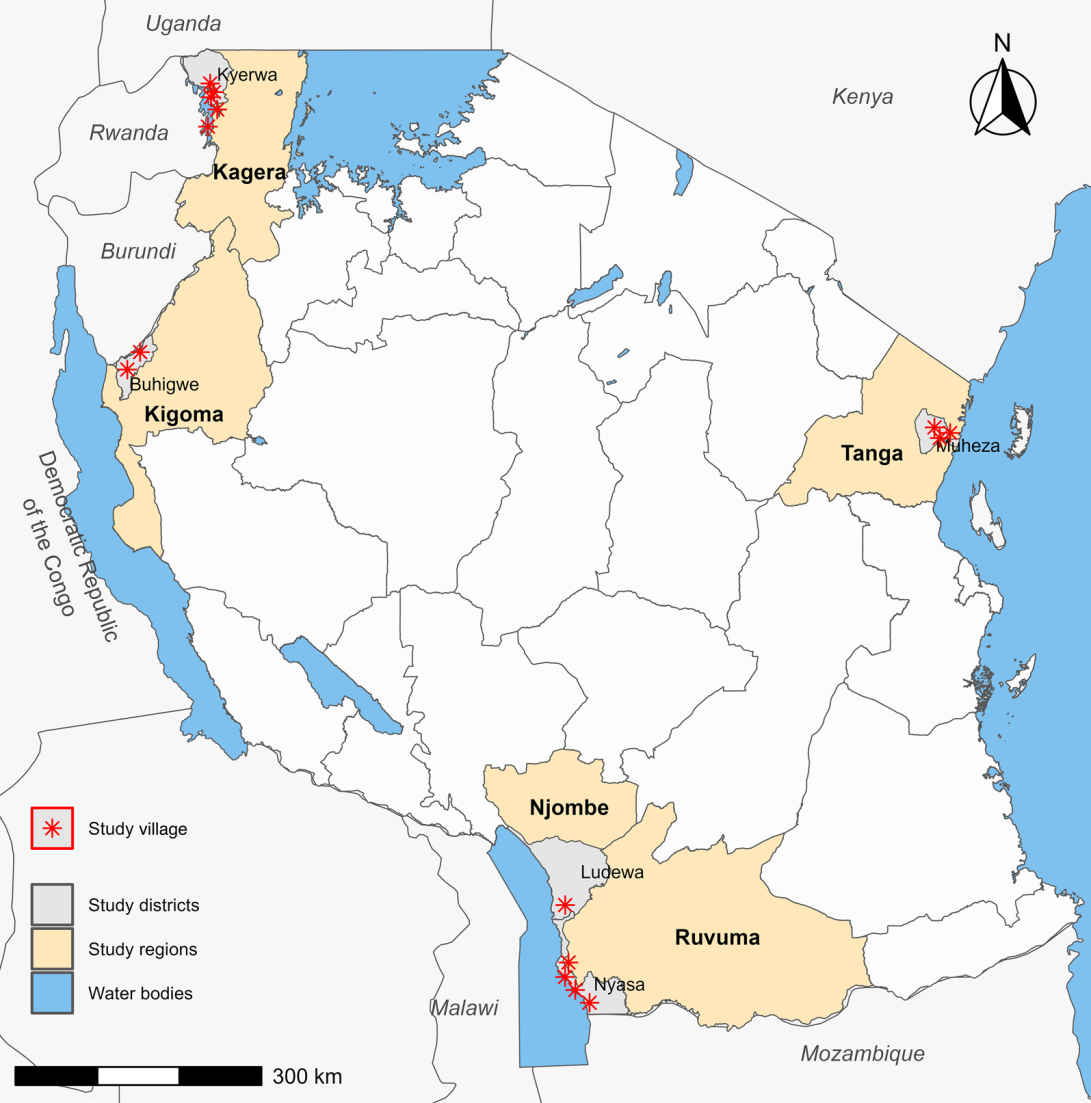
Map of Tanzania showing the study regions, districts and villages. This map was generated using the ggplot2 and sf packages in R software (version 4.41, R Core Team), and administrative boundary shapefiles were sourced from the Humanitarian Data Exchange (HDX), provided by GeoBoundaries (https://www.geoboundaries.org/), an open-license database of political administrative boundaries

This was a community-based CSS which was conducted in five regions of mainland Tanzania from July to August 2023. It was implemented as one of the components of the main project on molecular surveillance of malaria in Tanzania (MSMT). The MSMT project was implemented in 13 regions of mainland Tanzania from 2021 and 2022 and was later extended to cover all 26 regions from 2023 (D.S. Ishengoma, unpublished data). The five regions covered in this study have varying malaria transmission intensity. They include two regions from high (Kigoma and Ruvuma), two from moderate (Kagera and Tanga), and one region from low (Njombe) strata of malaria transmission (Fig. 1). In the Kagera region, the survey covered five villages from Kyerwa district as described in a recent work [28], and these are located in an area where recent reports have shown the emergence of parasites with ART-R [16, 17]. In Kigoma, the study was conducted in the two villages of Nyankoronko and Kigege (in Buhigwe district), and in Ruvuma, the surveys were undertaken in four villages (Chiulu, Lipingo, Lundo, and Ngindo) in Nyasa district as described previously [18, 29, 30]. In Njombe region, the surveys were conducted in Kipangala, which is a new village under the MSMT project’s longitudinal surveillance component aimed at monitoring the trend and pattern of parasite populations (D.S. Ishengoma, unpublished data) to support the capture and tracking of parasites with *hrp2/3* gene deletions which were recently detected in the area [18]. In Tanga region, the survey was undertaken in the three villages of Magoda, Mamboleo, and Mpapayu, all located in Muheza district, where different surveys were conducted beginning in 1992, and the community has been under surveillance ever since, as reported elsewhere [31–33].

### Study population and recruitment of participants

This study was conducted within the communities under the CSS and longitudinal health facility follow-up components of the MSMT project and targeted all individuals aged ≥ 6 months living in the study villages who were registered during the census surveys undertaken by the MSMT project before the CSS as described previously [28]. Individuals were recruited upon meeting the inclusion criteria which included age ≥ 6 months, residence in one of the study villages and provision of informed consent. Convenient sampling was employed, and study participants were enrolled based on their willingness to visit the recruitment posts and provide informed consent to take part in the CSS, with the aim of recruiting 20–30% of the members of each village. Based on experience from previous community surveys undertaken by the team from the Tanzanian National Institute for Medical Research (NIMR), this sample size is a better representation of all members of the village and was manageable given the limited resources and time allocated for the survey [28]. The study did not include individuals from villages not covered by the MSMT project or those who declined to provide informed consent. All community members were informed about the study by the community/village/hamlet sensitization teams, which passed the information to the community using a loudspeaker for 1–2 days before the CSS. Each study village has 3–5 hamlets, and all registered members from each hamlet were invited to meet the study team at the CCS post on designated survey days. The survey was conducted in each village for 2–3 days, and on each day, 1–2 hamlets were invited to visit the CSS post which was established in one of the hamlets. Household members who failed to attend on their scheduled day(s) were allowed to visit the study team on any other day in the same village or even in a nearby village in cases where the survey covered more than one village in the same area.

### Data collection procedures

Prior to the CSS, census surveys were conducted in each village to collect demographic data of community members, register households, and obtain household socio-economic status (SES) and environmental, land-use, and geographical positioning system (GPS) data according to the procedures of the MSMT project [28]. Members of the selected villages in each region and their households were enumerated and given unique identification numbers (IDs) as described previously [28]. During the CSS, all participants were identified using their unique IDs that were given during the census, and data collection was conducted according to the standard procedures of the MSMT project [28]. For the CSS, four different sections were used for (1) identification, registration, and collection of socio-demographic data, (2) collection of anthropometric measurements (height, weight and axillary temperature), (3) conducting a clinical assessment of each participant, and (4) performing parasitological tests with RDTs as well as collecting blood samples for different laboratory assays. Briefly, each participant reported to the registration section and had their identities verified and were assigned study IDs specific to the CSS and provided with registration cards. Study procedures in the first section included obtaining consent and/or assent (for participants aged 7–17 years), and interviews to collect socio-demographic data and the use of malaria prevention interventions. Participants were directed to the next section for anthropometric measurements (body weight, height, and axillary temperature) and then to the laboratory section where finger pricks was done for the detection of malaria parasites using RDTs and collection of blood smears for microscopy and dried blood spots (DBS) on filter papers (Whatman no. 3, GE Healthcare Life Sciences, PA, USA). The RDTs which were used in the CSS included Abbott Bioline Malaria Ag Pf/Pan (Abbott Diagnostics Korea Inc., Gyeonggi-do, Korea) and Malaria Pf/Pan Ag Rapid Test (Zhejiang Orient Gene Biotech Co., Ltd., Zhejiang, China). From the same finger prick, DBS and blood smears (thin and thick) were also collected for further laboratory analyses (processing and analysis of samples is underway and data will be reported in our future reports). The final section was for clinical assessment, where participants were assessed by study clinicians to obtain data on their history of illness and any treatment taken within 7 days before the survey. Each participant had a physical examination and clinical diagnosis, and those who had a positive malaria test or any other illness were managed according to the national guidelines for the treatment of malaria [34] or the guidelines for other febrile conditions [35].

### Data management and analysis

Data collection was performed using structured questionnaires which were developed and configured to run on Open Data Kit (ODK) software and installed on tablets. Subsequently, the collected data were transferred daily to the central server at NIMR in Dar es Salaam. Data quality checks were integrated into the databases through consistency checks, whereby any unexpected values were flagged for rejection, prompting corrections by the field team. The data were exported to Excel for additional cleaning, which was performed daily during the CSS and after completion of the survey. The data were later transferred to Stata version 13 (StataCorp LLC, College Station, TX, USA) for final cleaning and analysis. Initial descriptive analysis was performed to provide baseline information on the study populations and their demographic characteristics. The Chi-square test was used to assess the relationships between categorical variables. The results are presented in tables, figures, and text.

Multilevel logistic regression was used to assess the association between malaria infection and other covariates including age group [which included under-fives, school children (aged 5-9 and 10-14) and adults aged ≥15 years], sex, household size, education level, and geographical location. Variables with *P* < 0.25 in the univariate analysis were fitted into multivariate models. Hierarchical model-building strategies were used for further analysis, whereby the first model was generated by adjusting for individual-level variables such as sex, age group, and the use of bednets the night before the survey. In the second (model II), the analysis included both individual and household characteristics such as household size, household wealth index, and the type of house inhabited (focusing on the type of windows and walls and presence/absence of eaves). Principal component analysis (PCA) was used to determine the SES of the households based on the assets owned by each family, using the data collected during the census survey to compute the wealth index of the families in the study villages as described previously [28]. The variables included in the PCA were household possessions and assets such as the occupation of the head of household, number of rooms in the house, ownership of items such as mobile phones, motorcycles, bicycles, and domestic animals such as cattle, goats, chickens, and pigs. Other assets included land owned by the family and the number of acres cultivated, the source of drinking water, lighting and cooking energy, and the type of toilet. The scores of the first component with eigenvalues > 1 were used to create the household wealth index/SES (categorized as high, moderate or low). The inter-cluster correlation coefficient (ICC) was used to estimate the proportion of variance contributed to household characteristics, and other parameters such as the Akaike information criterion (AIC) and log-likelihood test (−2LL) tests were used to assess the goodness of fit. The association between variables was reported as crude (cOR) or adjusted odds ratios (aOR) with 95% confidence intervals (CIs), and a *P*-value ≤ 0.05 was considered statistically significant.

## Results

### Baseline demographic characteristics of participants

The study covered 15 villages from the five districts of Buhigwe (Kigoma region, with two villages), Kyerwa (Kagera, with five villages), Ludewa (Njombe, one village), Muheza (Tanga region with three villages), and Nyasa (Ruvuma, four villages). The study villages had a population of 40,116 individuals residing in 8881 households. Of the entire population in these villages, 10,228/40,116 (25.5%) were recruited in the CSS, which was conducted in July and August 2023 (Table 1). Most of the study participants were female (60.3%, *n* = 6163/10,228), and the proportions of participants of different sex varied significantly in the different districts (*P* = 0.006). Most of the participants (47.9%, *n* = 4897/10,228) were aged ≥ 15 years, and the overall median age was 14.1 years (interquartile range [IQR] = 7.2–38.1 years), with significant differences among the districts (the median age ranged from 11.9 years in Buhigwe to 17.6 years in Ludewa, *P* < 0.001). A high proportion of the participants (34.1%, *n* = 2451/10,228) had completed primary education, and their main occupation was farming, which was reported by 2568/10,228 (39.8%) participants. Of all recruited participants, 2102/10,228 (20.6%) had a history of fever in the last 48 h before the survey, and only 238/10,228 (2.3%) individuals had fever at presentation (with an axillary temperature ≥ 37.5 °C) (Table 1).

**Table 1:** Demographic characteristics of study participants

Variable	Total	Ludewa	Buhigwe	Muheza	Kyerwa	Nyasa	*P*-value
Number of households	8881	390	998	945	4155	2393	
Number of individuals^a^	40,116	1604	5763	5310	17,519	9920	
Enrolled, *N*	10,228	611	1453	1255	4454	2455	
Sex, *n* (%)							
Female	6163 (60.3)	362 (59.2)	914 (62.9)	720 (57.4)	2641 (59.3)	1526 (62.2)	0.006
Male	4065 (39.7)	249 (40.8)	539 (37.1)	535 (42.6)	1813 (40.7)	929 (37.8)	
Age in years, median (IQR)	14.1 (7.2–38.1)	14.1 (7.1–43.1)	11.9 (6.5–26.1)	14.2 (8.3–39.2)	14.2 (6.7–36.0)	17.6 (8.6–45.1)	< 0.001
Age group, *n* (%)							
< 5 years	1692 (16.5)	96 (15.7)	261 (18.0)	162 (12.9)	835 (18.8)	338 (13.8)	< 0.001
5–9 years	1849 (18.1)	120 (19.6)	325 (22.4)	239 (19.1)	772 (17.3)	393 (16.0)	
10–14 years	1789 (17.5)	99 (16.2)	347 (23.9)	251 (20.0)	700 (15.7)	392 (16.0)	
≥ 15 years	4897 (47.9)	296 (48.5)	521 (35.8)	601 (48.0)	2147 (48.2)	1332 (54.3)	
Education, *n* (%)	7186	476	995	688	3249	1778	
None	865 (12.0)	14 (2.9)	92 (9.2)	43 (6.3)	630 (19.4)	86 (4.8)	< 0.001
Incomplete primary	748 (10.4)	43 (9.0)	72 (7.2)	64 (9.3)	375 (11.5)	194 (10.9)	
Primary	2451 (34.1)	202 (42.4)	266 (26.7)	264 (38.4)	940 (28.9)	779 (43.8)	
Secondary or above	221 (3.1)	21 (4.4)	10 (1.0)	28 (4.1)	75 (2.3)	87 (4.9)	
Studying	2901 (40.4)	196 (41.2)	555 (55.8)	289 (42)	1229 (37.8)	632 (35.5)	
Occupation, *n* (%)	6452	555	374	100	3944	1479	
Farmer	2568 (39.8)	233 (41.9)	60 (16.0)	6 (6.1)	1636 (41.5)	632 (42.7)	< 0.001
Student	1855 (28.8)	155 (28.0)	137 (36.6)	32 (32.7)	1061 (26.9)	469 (31.7)	
Child	1846 (28.6)	147 (26.5)	160 (42.8)	52 (53.1)	1183 (30.0)	305 (20.6)	
Others	183 (2.8)	20 (3.6)	17 (4.6)	8 (8.2.0)	64 (1.6)	73 (4.9)	
Ownership of bednet, *n* (%)							
Yes	7939 (77.6)	555 (90.8)	1101 (75.8)	1154 (92.0)	2867 (64.4)	2262 (92.0)	< 0.001
No	2289 (22.4)	56 (9.2)	352 (24.2)	101 (8.0)	1587 (35.6)	193 (8.0)	
Slept under bednet last night, *n* (%)							
Yes	7899 (77.2)	555 (90.8)	1094 (75.3)	1145 (91.2)	2849 (64.0)	2256 (91.9)	< 0.001
No	2329 (22.8)	56 (9.2)	359 (24.7)	110 (8.8)	1605 (36.0)	199 (8.1)	
History of fever^b^, *n* (%)							
Yes	2102 (20.6)	72 (11.8)	338 (23.3)	246 (19.6)	1342 (30.1)	104 (4.2)	< 0.001
No	8126 (79.4)	539 (88.2)	1115 (76.7)	1009 (80.4)	3112 (69.9)	2351 (95.8)	
Measured fever^c^, *n* (%)							
Yes	238 (2.3)	5 (0.8)	25 (1.7)	21 (1.7)	136 (3.0)	51 (2.1)	< 0.001
No	9990 (97.7)	606 (99.2)	1428 (98.3)	1234 (98.3)	4318 (97.0)	2404 (97.9)	
BW (kg), median (IQR)	36 (19–51)	36 (18–54)	28 (17–46)	38 (22–53)	36 (18–52)	43 (23–53)	< 0.001
Height (cm), median (IQR)	145 (115–157)	142 (113–156)	134 (108–152)	148 (125–159)	145 (110–158)	146 (121–156)	< 0.001
Temp (°C), mean (SD)	36.1 (0.8)	35.6 (0.9)	36.1 (0.7)	36.4 (0.4)	36.1 (0.9)	36.1 (0.8)	< 0.001

*N* population size, *n* number of participants, *BW* body weight, *IQR* interquartile range, *Temp* temperature, *SD* standard deviation

^a^Total population in the study communities

^b^History of fever in the past 2 days

^c^Fever measured at presentation and denoted as axillary temperature ≥ 37.5 °C

### Prevalence of malaria infection by rapid diagnostic tests

Among all districts, 3515/10,228 (34.4%) participants had positive results by RDTs. The lowest prevalence (21.6%, *n* = 271/1255) was in the three villages of Muheza (Tanga region) while the highest was in the five villages of Kyerwa district in the Kagera region (44.4%, *n* = 1979/4454), and the differences in prevalence among the districts were statistically significant (*P* < 0.001). The prevalence was significantly higher in males (38.9%, *n* = 1583/4065; *P* < 0.001) than in females (31.3%, *n* = 1932/6163), and in schoolchildren [aged 5–9 years (44.6%, *n* = 828/1855; *P* < 0.001) and aged 10–14 years (47.2%, *n* = 843/1785; *P* < 0.001)] compared to children under 5 years (38.3%, *n* = 646/1689) and adults aged ≥ 15 years (24.5%, *n* = 1198/4899). The prevalence was also significantly higher among participants who did not use bednets the night before the survey (41.5%, *n* = 965/2,328; *P* < 0.001) than in bednet users (32.2%, *n* = 2547/7900), and in those who reported a history of fever in the past 2 days (77.4%, *n* = 1626/2102; *P* < 0.001) or had fever at presentation (62.6%, *n* = 149/238; *P* < 0.001). Children under 5 years from all districts had lower prevalence than schoolchildren but higher prevalence than adults (≥ 15 years old) (Table 2).

**Table 2:** Prevalence of malaria infections in the five districts

Variable	Ludewa	Buhigwe	Muheza	Kyerwa	Nyasa	Total
Total enrolled, *N*	611	1453	1255	4454	2455	10,228
RDTs positive, *n* (%)	215/611 (35.2)	482/1453 (33.2)	271/1255 (21.6)	1979/4454 (44.4)	568/2455 (23.1)	3515/10,228 (34.4)
Sex, *n* (%)						
Female	113/362 (31.2)	273/914 (29.9)	134/720 (18.6)	1089/2641 (41.2)	323/1526 (21.2)	1932/6163 (31.3)
Male	102/249 (41.0)	209/539 (38.8)	137/535 (25.6)	890/1813 (49.1)	245/929 (26.4)	1583/4065 (38.9)
*P*-value	0.013	< 0.001	0.003	< 0.001	0.003	< 0.001
Age group, *n* (%)						
< 5 years	32/96 (33.3)	78/260 (30.0)	21/160 (13.1)	452/835 (54.1)	63/338 (18.6)	646/1689 (38.3)
5–9 years	62/120 (51.7)	119/325 (36.6)	61/243 (25.1)	472/773 (61.1)	114/394 (28.9)	828/1855 (44.6)
10–14 years	60/99 (60.6)	145/347 (41.8)	78/250 (31.2)	400/698 (57.3)	160/391 (40.9)	843/1785 (47.2)
≥ 15 years	61/296 (20.6)	140/521 (26.9)	111/602 (18.4)	655/2148 (30.5)	231/1332 (17.3)	1198/4899 (24.5)
*P*-value	< 0.001	< 0.001	< 0.001	< 0.001	< 0.001	< 0.001
Bednet ownership, *n* (%)						
Yes	195/555 (35.1)	350/1101 (31.8)	247/1154 (21.4)	1262/2867 (44)	517/2262 (22.9)	2557/7940 (32.2)
No	20/56 (35.7)	132/352 (37.5)	24/101 (23.8)	717/1587 (45.2)	51/193 (26.4)	945/2288 (41.3)
*P*-value	0.931	0.048	0.581	0.455	0.259	< 0.001
Slept under the bednet last night, *n* (%)						
Yes	195/555 (35.1)	349/1094 (31.9)	242/1145 (21.1)	1258/2849 (44.2)	517/2256 (22.9)	2547/7900 (32.2)
No	20/56 (35.7)	133/359 (37.1)	29/110 (26.4)	721/1605 (44.9)	51/199 (25.6)	965/2328 (41.5)
*P*-value	0.931	0.072	0.203	0.621	0.385	< 0.001
History of fever^a^ *n* (%)						
Yes	61/72 (84.7)	173/338 (51.2)	150/246 (61.0)	1187/1342 (88.5)	55/104 (52.9)	1626/2102 (77.4)
No	154/539 (28.6)	309/1115 (27.7)	121/1009 (12.0)	792/3112 (25.5)	513/2351 (21.8)	1889/8126 (23.2)
*P*-value	< 0.001	< 0.001	< 0.001	< 0.001	< 0.001	< 0.001
Measured fever^b^, *n* (%)						
Yes	4/5 (80.0)	12/25 (48.8)	13/21 (61.9)	108/136 (79.4)	12/51 (23.5)	149/238 (62.6)
No	211/606 (34.8)	470/1428 (32.9)	258/1234 (20.9)	1871/4318 (43.3)	556/2404 (23.1)	3366/9990 (33.7)
*P*-value	0.035	0.112	< 0.001	< 0.001	0.946	< 0.001

*N* population size, *RDTs* rapid diagnostic tests, *n* number of participants

^a^History of fever in the past 2 days

^b^Fever measured at presentation, characterized as axillary temperature ≥ 37.5 °C

**Fig. 2:**
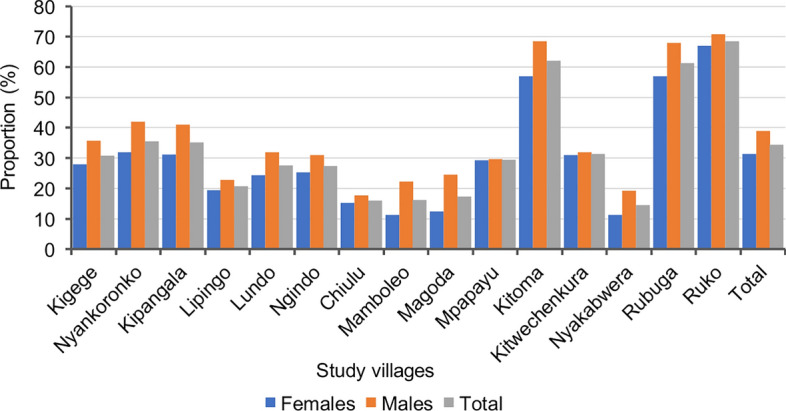
Prevalence of malaria infection by RDTs among male and female participants from different villages in the five districts (I would recommend indicating districts and regions where these villages are located; Kigege and Nyankoronko - Buhigwe - Kigoma; Kipangala - Ludewa - Njombe; Lipingo, Lundo, Ngindo and Chiulu - Nyasa - Ruvuma; Mamboleo, Magoda and Mpapayu - Muheza, Tanga; and Kitoma, Kitwechenkura, Nyakabwera, Rubuga and Ruko - Kyerwa, Kagera)

Malaria prevalence varied significantly among villages in some districts (*P* < 0.001). Villages in Kyerwa district had higher malaria prevalence than other districts, ranging from the lowest of 14.5% in Nyakabwera to the highest of 68.5% in Ruko village, while villages in Muheza had the lowest overall prevalence, ranging from 16.3% in Mamboleo to 29.0% in Mpapayu village. Statistically significant differences in malaria prevalence were observed in villages within Muheza, Kyerwa, and Nyasa districts (*P* < 0.001), but no significant difference was observed between the two villages in Buhigwe district (Nyankoronko and Kigege,* P* = 0.056). Across all districts, only four villages had malaria prevalence below 20.0%, including two in Muheza (Mamboleo and Magoda) and one village each in the districts of Kyerwa (Nyakabwera) and Nyasa (Chiulu); the highest heterogeneity was observed in Kyerwa (Fig. 2 and Table 2).

**Fig. 3:**
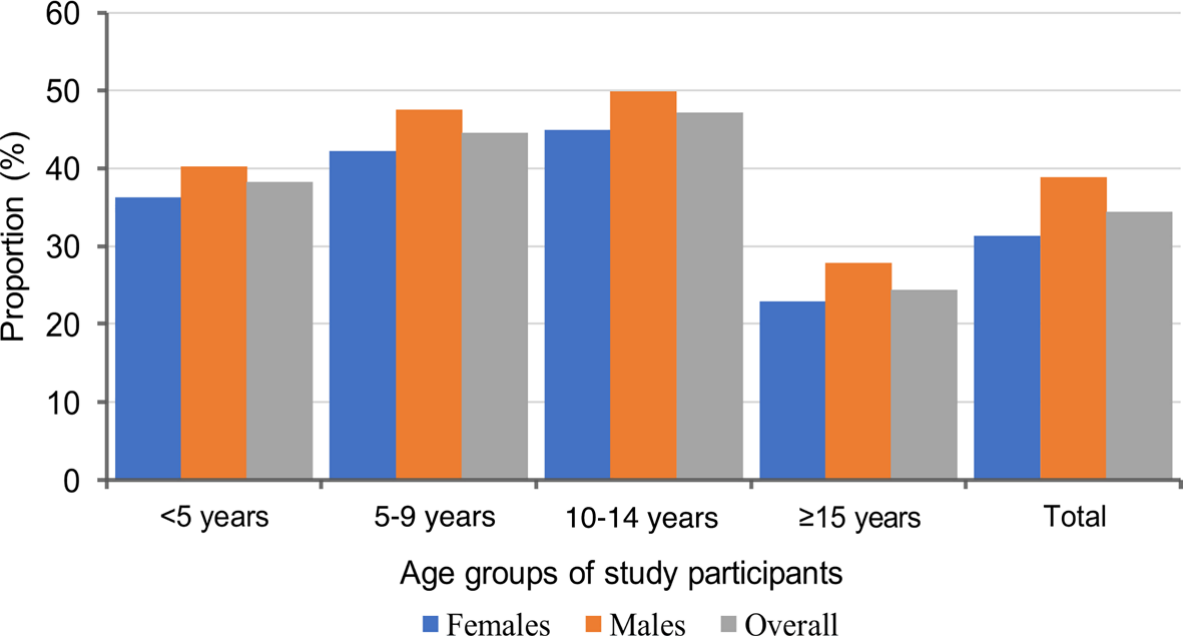
Prevalence of malaria infection by RDTs among male and female participants of different age groups

**Fig. 4:**
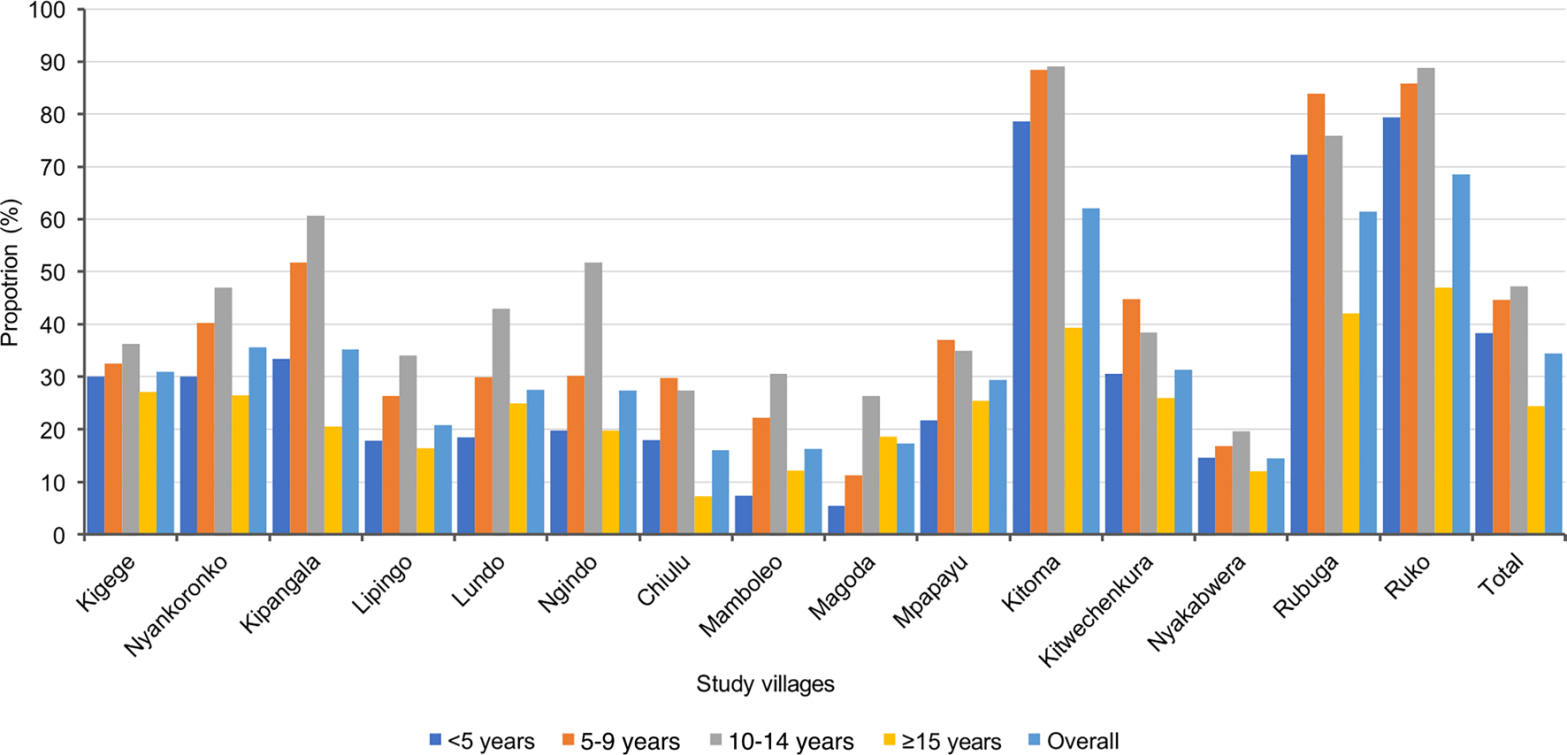
Prevalence of malaria infection among individuals of different age groups in the study village from the five districts (see my recommendation of including the districts and regions given on Fig. 2)

Among the different age groups, schoolchildren had the highest prevalence of malaria infections, with those aged 10–14 years (47.2%) and 5–9 years (44.6%), followed by those under 5 years (38.3%), and the lowest prevalence (24.5%) was observed in adults aged ≥ 15 years (Fig. 3). Across villages, the prevalence was higher among schoolchildren than other age groups. Conversely, adults in the villages had the lowest prevalence except for the three villages from Muheza district (Mamboleo, Magoda and Mpapayu), where children under five years had the lowest prevalence among groups. With the exception of four villages (Chiulu in Buhigwe, Mpapayu in Muheza, and Kitwechenkura and Rubuga in Kyerwa), the highest prevalence of malaria infections was observed in older schoolchildren (aged 10–14 years) (Fig. 4 and Table 2). Malaria prevalence was higher among males than females in all villages (Fig. 2). In children under five years, the pattern of prevalence in males and females differed among the study villages, where some villages had higher prevalence in males, and in other villages the prevalence was higher in females. In schoolchildren, the prevalence was higher in males in all villages with the exception of the six villages of Chiulu (Nyasa district, Ruvuma), Nyankoronko (Buhigwe, Kigoma), Mpapayu (Muheza, Tanga), and Kitwechenkura, Kitoma, and Ruko (Kyerwa, Kagera region) (Additional file 1: Fig. S1).

### Bednet ownership and use, and prevalence of malaria infection

**Fig. 5:**
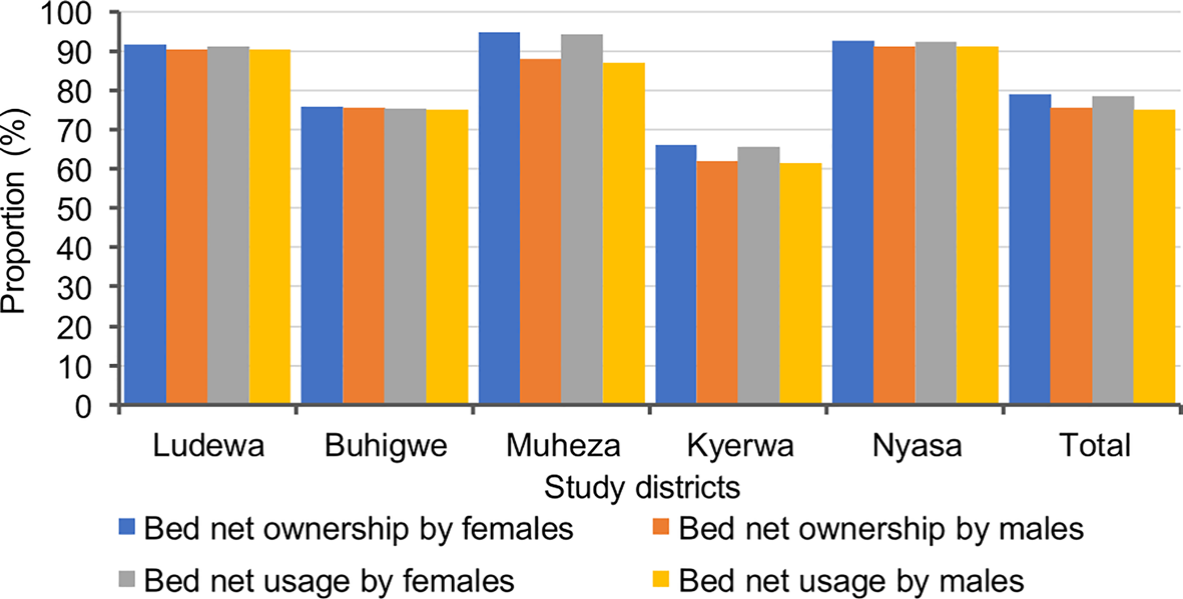
Bednet ownership and usage by sex in the five districts (ownership and usage of bednets were reported by adult respondents or parents/caretakers of children aged less than 18 years)

**Fig. 6:**
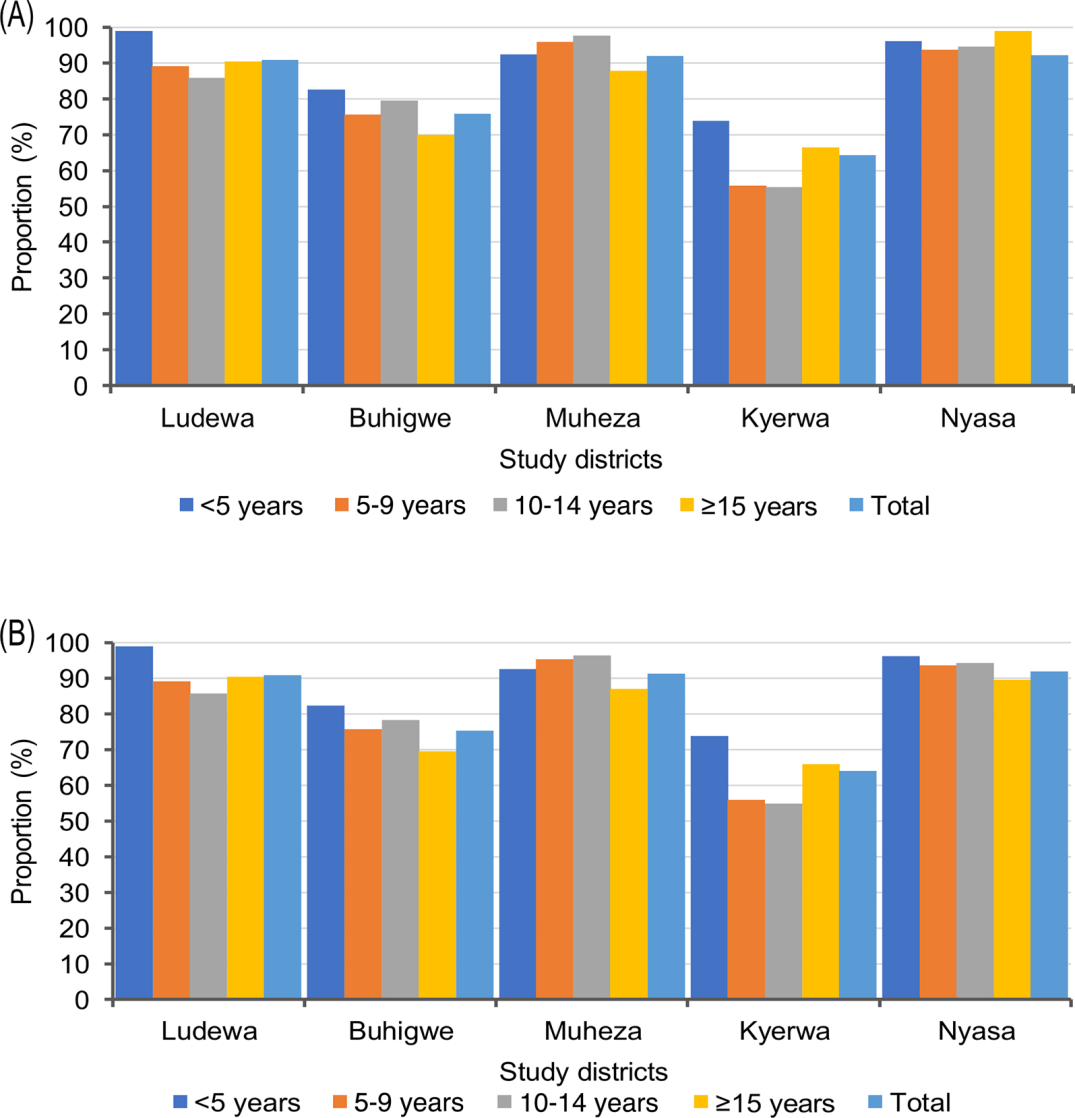
Bednet ownership (**A**) and use (**B**) among individuals of different age groups in the five districts (ownership and usage of bednets were reported by adult respondents or parents/caretakers of children aged less than 18 years)

About 78.0% of the participants (*n* = 7939/10,228) had bednets, and 77.2% (*n* = 7899/10,228) had slept under the nets the night before the survey (Table 1). Bednet ownership was significantly higher (≥ 91.0%) in the three districts of Ludewa, Muheza, and Nyasa than in Buhigwe (75.8%) and Kyerwa (64.4%), which had lower ownership of nets. A similar trend was observed in bednet usage, with the rates exceeding 91.0% in the three districts of Ludewa, Muheza, and Nyasa, and lower rates of 75.3% in Buhigwe and 64.0% in Kyerwa (*P* < 0.001 for all comparisons). Bednet ownership and use were significantly higher among females in the districts of Kyerwa (*P* ≥ 0.005) and Muheza (*P* < 0.001), while it was similar between male and female participants in the other three districts (Buhigwe, Ludewa, and Nyasa; *P* ≥ 0.218) (Fig. 5**)**. In all age groups, children under 5 years had higher rates of bednet ownership across all districts except Muheza. Schoolchildren in Ludewa and Kyerwa, and adults in Buhigwe and Nyasa had lower rates of bednet ownership. In Muheza district, bednet ownership was higher among schoolchildren, followed by those under 5 years, and remained lower in adults (Fig. 6A). Similarly, bednet usage was notably higher among children under 5 across all districts except Muheza. Usage of nets was lower among schoolchildren in Ludewa and Kyerwa and among adults in Buhigwe and Nyasa districts compared to other age groups. In Muheza district, bednet usage was higher in schoolchildren and lower among adults (*P* = 0.001) (Fig. 6B**)**.

A higher prevalence of malaria infection (over 41.0%) was observed among individuals who did not own or sleep under the nets compared to their counterparts (bednet owners and users). Higher prevalence was observed among participants from Kyerwa who neither owned (45.2%) nor slept under (44.9%) bednets. A similar trend was observed in all other districts. Overall, there was a significant association between malaria prevalence and bednet ownership as well as sleeping under bednets in the night before the survey (*P* < 0.001) (Table 2).

### Prevalence of malaria infection and household characteristics

The prevalence of malaria infection was significantly higher among individuals living in households with five or more people (35.4%) compared to those with fewer members (30.0%) (*P* < 0.001). Similarly, individuals from households with low SES had significantly higher malaria prevalence (37.9%) than those with moderate (33.3%) or higher SES (31.3%) (*P* < 0.001). The type of walls of the houses were also associated with malaria (*P* < 0.001), where individuals living in houses whose walls were made of mud exhibited a higher prevalence (37.1%) than those from houses constructed with bricks (32.9%). The presence of holes in the walls and the types of windows (closed, open and partially open) were significantly associated with malaria prevalence, where participants from households whose houses had holes in the walls (39.2%) and open windows (36.3%) had higher prevalence of malaria infections (Table 3).

**Table 3:** Prevalence of malaria infections by household characteristics

Variable	Examined (*N*)	Positive RDTs, *n* (%)	*P*-value
Family size			
< 5 people	2188	656 (30.0)	< 0.001
5 or more people	7476	2647 (35.4)	
SES			
Low	3259	1235 (37.9)	< 0.001
Moderate	3204	1067 (33.3)	
High	3201	1001 (31.3)	
Wall type			
Bricks	5794	1908 (32.9)	< 0.001
Mud	3236	1199 (37.1)	
Other	634	196 (30.9)	
Presence of holes in wall			
Yes	1074	421 (39.2)	< 0.001
No	8568	2876 (33.6)	
Window type			
Closed	3740	1182 (31.6)	< 0.001
Open	4869	1769 (36.3)	
Partially open	1030	346 (33.6)	

*N* population size, *n* number of participants, *RDTs* rapid diagnostic tests, *SES* socio-economic status

### Factors associated with the risk of malaria infection

The analysis performed using multivariate logistic regression after adjusting for both individual and household characteristics showed that the odds of malaria infection were higher in males (aOR = 1.32, 95% CI 1.19–1.48, *P* < 0.001) than females. In the different age groups, the odds of malaria infection were higher in children under 5 years (aOR = 2.02, 95% CI 1.74–2.40, *P* < 0.001) and schoolchildren [aged 5–9 years old (aOR = 3.23, 95% CI 2.78–3.76, *P* < 0.001) and 10–14 years (aOR = 3.53, 95% CI 3.03–4.11, *P* < 0.001)] than in adults. Higher odds of malaria infection (aOR 1.49; 95% CI 1.29–1.72, *P* < 0.001) were also observed in individuals who had not slept under bednets the night before the survey. In addition, individuals from households with low SES (aOR = 1.40, 95% CI 1.16–1.69, *P* < 0.001) and those from houses with open windows (aOR = 1.24, 95% CI 1.06–1.45, *P* < 0.001) and holes in the walls (aOR = 1.43, 95% CI 1.13–1.81, *P* < 0.001) had higher odds of malaria infection (Table 4).

**Table 4:** Socio-demographic and household characteristics associated with the risk of malaria infection among individuals enrolled from the five districts

Variables	uOR, 95% CI	*P*-value	aOR, 95% CI	*P*-value
Sex				
Male	1.57 (1.4–1.74)	< 0.001	1.32 (1.19–1.48)	< 0.001
Female (reference)	1		1	
Age groups				
< 5 years	2.15 (1.85–2.50)	< 0.001	2.02 (1.74–2.40)	< 0.001
5–9 years	3.45 (2.97–4.00)	< 0.001	3.23 (2.7–3.76)	< 0.001
10–14 years	3.70 (3.18–4.29)	< 0.001	3.53 (3.03–4.11)	< 0.001
≥ 15 (reference)	1		1	
Use of bednet in the previous night				
Yes (reference)	1		1	
No	1.62 (1.41–1.86)	< 0.001	1.49 (1.29–1.72)	< 0.001
Family size				
< 5 people (reference)	1		1	
5 or more people	1.28 (1.15–1.42)	< 0.001	1.11 (0.93–1.33)	0.17
SES				
Low	1.62 (1.41–1.86)	< 0.001	1.40 (1.16–1.69)	< 0.001
Moderate	1.36 (1.16–1.59)	0.08	1.16 (0.97–1.40)	0.11
High (reference)	1		1	
Window types				
Closed (reference)	1		1	
Open	1.30 (1.13–1.51)	< 0.001	1.24 (1.06–1.45)	< 0.001
Partially open	1.12 (0.88–1.41)	0.23	1.12 (0.87–1.43)	0.11
Wall type				
Bricks/blocks (reference)	1		1	
Mud	1.30 (1.12–1.50)	< 0.001	1.10 (0.94–1.30)	0.31
Other	0.92 (0.69–1.23)	0.57	0.83 (0.60–1.29)	0.24
Presence of holes in wall				
Yes	1.45 (1.17–1.81)	< 0.001	1.43 (1.13–1.81)	< 0.001
No (reference)	1		1	
Eaves				
Closed (reference)	1		1	
Open	0.89 (0.78–1.02)	0.1	1.09 (0.94–1.27)	0.24

*uOR* unadjusted odds ratio, *aOR* adjusted odds ratio, *CI* confidence intervals

The analysis of random effects measures revealed a significant clustering effect of the households, with ICC 0.33 indicating that about 33.0% of the total variation in malaria infection could be attributed to household characteristics (null model). The last model with the lowest AIC and likelihood ratio was considered the best model for predicting the association between independent variables and the prevalence of malaria (Table 5).

**Table 5:** Random-effects model and comparison of the best fit of factors associated with malaria prevalence

Parameter	Null model	Model I	Model II
Cluster-level variance (SE)	1.29 (0.15)	1.88 (0.15)	1.84 (0.14)
ICC	33.0%	36.0%	36.0%
Fit model criteria			
LL	−5892.54	−5628.98	−5606.18
AIC (−2LL)	11,785.072	11,258	11,212.4

*ICC* inter-cluster correlation coefficient, *AIC* Akaike information criterion, *LL* log-likelihood, *SE* standard error

## Discussion

Although malaria infections mainly involving asymptomatic individuals have been given little attention, there are increasing reports suggesting an urgent need to integrate and implement strategies targeting asymptomatic individuals in routine malaria control and elimination initiatives. This is due to the potential role of such asymptomatic infections, which act as a major reservoir of  malaria transmission, thus hindering progress to malaria elimination. Currently, there is limited information on the prevalence and potential drivers associated with asymptomatic infections which would provide evidence for targeting them with specific interventions particularly in the ongoing elimination efforts in Tanzania. This study was therefore conducted to assess the prevalence and drivers of malaria infection among community members (including both asymptomatic and symptomatic individuals) from selected communities in five regions of Tanzania with varying transmission intensity to support identification of high-burden areas and vulnerable groups.

In the current study, the overall prevalence of malaria infections was high (34.4%) and was highly variable at the community and regional levels. The study also showed high prevalence and risk of malaria infections among schoolchildren, males, participants with fever (as history in the past 2 days or fever at presentation), and those from households with many members, with low SES, and living in poorly constructed houses. The high prevalence of malaria reported in this study is comparable to what has been previously reported in Tanzania and elsewhere [28, 36, 37]. The high prevalence among community members (with or without symptoms of malaria) may be attributed to the presence of factors including vector harbouring or breeding sites [38], lack of proper knowledge of malaria prevention and control measures [39, 40], and other socio-economic-related factors resulting in failure to afford better houses and interventions that reduce malaria transmission.

This study reported a higher malaria prevalence in males than females (in all age groups except in a few cases where the prevalence was higher in females of some age groups), and this is consistent with other findings reported elsewhere [28, 41]. It was also shown that males had higher odds of malaria infection than females, possibly due to the lifestyle and activities of males living in rural communities. This study and previous studies conducted elsewhere showed that males are usually more involved in socio-economic activities such as agriculture, fishing, and grazing in environments that are suitable for mosquito breeding [42, 43]. It has also been reported that males spend most of their time outdoors in social gatherings and cultural events up to peak biting hours, in contrast to females, increasing their exposure to mosquito bites [44, 45]. Males are also less likely than females to seek medical care when they experience malaria-related symptoms, increasing their vulnerability to malaria infections [46–48]. In addition to behavioural and socio-economic factors, there are sex-specific biological factors, such as post-pubertal hormone changes in males (aged ≥ 15 years), that increase their allure to mosquitoes. Immunity-related factors may also contribute to the increased prevalence of malaria infections in males [49, 50]. Studies have suggested that males experience a delayed clearance of infection compared to females in the absence of treatment [51, 52]. As a result, males tend to remain asymptomatic for a longer period, increasing their likelihood of testing positive during surveys. These findings suggest that more studies are needed to fully uncover the reasons and causes of higher prevalence of malaria infections in males, and support the development of methods to target them with appropriate interventions.

Schoolchildren had a higher prevalence above the national average of 21.6%, as reported elsewhere [53, 54]. The odds of being infected with malaria parasites was three times as high among schoolchildren as among adults. The higher odds in this group may be attributed to their involvement in risky activities such as studying at night in areas where they are not protected against mosquito bites, traditional initiation ceremonies, and attending social events occurring outdoors up to late at night, which expose them to mosquito bites [54, 55]. Furthermore, the relatively higher odds of malaria infections among schoolchildren may be attributed to a recent epidemiological shift in the peak burden of asymptomatic malaria infections which previously occurred in children under 5 years [56, 57]. This shift in the peak malaria prevalence from children under 5 to schoolchildren (especially those of older age, from 10 to 15 years) has been attributed to the epidemiological transition of malaria. This change is largely due to the scaled-up and targeted interventions to infants and pregnant women (generally considered as vulnerable groups) such as ITNs, IPTp, and IPTi, and improved case management through timely diagnosis and treatment [33, 58, 59]. Thus, more interventions targeting schoolchildren are still needed to reduce the burden of malaria in this group, given the persistently higher burden of infection as shown by other studies conducted in Tanzania in recent years [28, 60–62].

In this study, children under 5 years had higher malaria prevalence than adults and their odds of being infected were twice as high as those of adults. However, the prevalence of malaria in children under 5 was lower than that of schoolchildren, consistent with other studies and attributed to the recent interventions which have reduced the transmission intensity, leading to delayed development of acquired immunity to malaria [60, 63]. The high prevalence of malaria infection in this group may be because children under 5 in areas with intense malaria transmissions are still exposed to infectious mosquito bites at an early age, leading to the development of naturally acquired immunity which enables them to carry parasites at lower levels, without symptoms of malaria [63, 64]. Additionally, the higher odds of infection in this group could be attributed to reduced attention from mothers and caregivers as children grow older, making them more vulnerable to mosquito bites, and consequently to malaria infections [65]. Hence, control efforts targeting children under 5 should continue in particular in areas where malaria transmission is persistently high, since the risk is still high.

Among all studied villages in the five districts, three villages (Rubuga, Kitoma, and Ruko) in Kyerwa district had a higher prevalence (> 61.0%) relative to the others. Conversely, only four villages had a prevalence of less than 20.0%, including two villages (Magoda and Mamboleo) from Muheza district and one village each from the districts of Nyasa (Chiulu) and Kyerwa (Nyakabwera). The prevalence varied significantly across villages, especially in Kyerwa district, where villages that are proximate to each other experienced different levels of malaria burden, suggesting the existence of other potential environmental factors contributing to the micro-geographical pattern of malaria within these communities. For instance, all study villages in Kyerwa (except Nyakabwera) are partly bordered by small lakes [28] which are part of the Kagera river basin, and these provide suitable breeding sites for malaria vectors [66, 67]. The variation in malaria burden at the micro-geographical level was also observed in another study conducted in Tanzania [68], which revealed heterogeneity in the risk of malaria among 80 councils. The reasons for low malaria prevalence in villages from other districts could include the efforts by the government through the NMCP to implement effective malaria control interventions, including free ITN distribution campaigns, IPTp, and strengthened malaria surveillance systems in areas with high transmission [25, 69, 70]. However, further studies are still needed to enable an understanding of the patterns and causes of heterogeneity in malaria at the micro-geographical level, and the development of interventions to target hotspots of malaria as part of the ongoing elimination strategies in Tanzania.

The distribution and use of bednets constitute the core interventions for prevention and control of malaria in Tanzania [1, 2]. The nets offer protection against mosquito bites, effectively reducing the transmission of malaria parasites by mosquitoes and therefore contributing to a significant decrease in malaria risk and burden at both the individual and community levels [22, 71, 72]. In this study, the overall bednet ownership and use the night before the survey were 77.6% and 77.2%, respectively. This is higher than that reported elsewhere [1, 73]. Bednet ownership and use were significantly higher (≥ 91.0%) in three districts, namely Muheza, Ludewa, and Nyasa, compared to others. The higher rates of ownership and bednet use are likely the result of extensive ITN distribution efforts that have been intensively undertaken by  the NMCP in the past two decades [74]. In Tanzania, NMCP aimed at ensuring universal access to and use of ITNs at a rate of at least one ITN for every two people and reaching coverage of 80% by 2023 and 85% by 2025 [2]. According to the 2022 national malaria indicator surveys, 74.0% of surveyed households reported owning at least one mosquito net [1]. Additional studies are needed to uncover factors contributing to reduced bednet ownership and usage in Kyerwa (Kagera) and Buhigwe districts (Kigoma region), and possibly other parts of Tanzania, and to develop strategies to reach the 2025 national targets.

Bednet ownership and use were higher among females and children under 5 years across districts, and this is not surprising, as there is an ongoing campaign in Tanzania focused on providing free bednets to pregnant women during antenatal care visits and children at 9 months during measles vaccination [75]. This is to ensure availability of ITNs for protection against malaria for both expectant mothers and their unborn children [70, 76], and for protection of children under 5 from malaria infections [75]. Likewise, schoolchildren have been targeted for controlling and reducing malaria burden in Tanzania for a decade now, and a school bednet programme has been active in schools to sustain ITN access and use since 2013 [77, 78]. Several studies have reported increased bednet ownership among schoolchildren in Tanzania [79, 80]. Despite the increase in ownership and use rates of bednets among schoolchildren, reaching 75.5% and 75.2%, respectively, this group still exhibited a high prevalence of malaria infections. Further studies are needed to assess different questions related to bednet ownership and use among schoolchildren as well as the reason for the high malaria burden in this group.

A significantly higher prevalence of malaria infections was observed in families with five or more members, those with low SES, and those living in houses constructed with mud compared to their counterparts. Furthermore, the presence of holes in the walls and open windows in the houses were associated with a higher prevalence and risk of malaria infections. These findings align with other studies indicating increased malaria vector biting risk with a higher number of household occupants in rural communities. This could be due to increased likelihood of transmission if one household member is infected with malaria and acting as a parasite reservoir, leading to the rapid spread to other members of the household [81]. Low SES is also associated with high risk of malaria infection, as it decreases the ability to afford malaria prevention, control, and treatment services and the ability to afford better houses, as well as contributing to low levels of knowledge regarding malaria control at the individual and household levels [82–84]. Additionally, the mud-constructed houses with openings and unscreened windows provide entry points for mosquitoes and consequently result in higher indoor vector density, which increases the risk of malaria transmission [72, 81]. Other studies have similarly reported a decreasing risk of malaria transmission associated with high-quality housing [85, 86]. Therefore, enhanced awareness of better housing and interventions that reduce malaria transmission is critical. It is also critical to devise and facilitate financial initiatives for poverty reduction in order to improve SES and housing conditions, which will in turn support vector control and expedite malaria elimination efforts.

Individuals who reported not sleeping under bednets the night before the survey had higher malaria prevalence, in line with what was reported by other studies conducted elsewhere [28, 87, 88]. The risk of malaria infection in this group was 49.0% higher than their counterparts. Various studies have shown that sleeping under ITNs offers a physical barrier to mosquito bites and effectively prevents malaria transmission by killing or deterring mosquitoes [71, 89]. Therefore, educational initiatives are needed to enable rural communities to understand the critical roles played by ITNs against malaria transmission [90, 91].

The current study found a negative correlation between SES and malaria burden. The risk of malaria among individuals with low SES was 40.0% higher than those with higher SES, and this is consistent with findings from studies reported elsewhere [82, 92, 93]. This increased risk could be attributed to various factors, including limited access to healthcare services and preventive measures such as ITNs and IRS [83, 94, 95]. Most individuals with low SES reside in poorly constructed houses with holes in the walls and unscreened windows, and these are associated with increased indoor mosquito bites, significantly increasing their vulnerability to malaria infections [96, 97]. Studies have suggested implementing different house modifications to reduce the risk of malaria transmission, including screening windows and doors, repairing walls, and using modern roofing materials. Thus, devising strategies aimed at addressing SES inequalities [85, 98, 99] and improving housing conditions in rural communities is critical to accelerating malaria control and eventually elimination.

Limitations of this study include the use of RDT-based results only, which are less sensitive than molecular approaches, particularly polymerase chain reaction (PCR), which would allow us to detect and account for the proportion of individuals with false-negative and false-positive results, which are common when using RDTs [100]. Thus, future studies should address the issue and help to determine the best test to use in the surveillance of asymptomatic malaria infection. The surveys which generated the data for this study were carried out during the dry season, when malaria transmission in some regions is low [101, 102], which might have resulted in an underestimation of overall malaria prevalence. The use of convenience sampling in the CSS may have introduced bias, since participants may have enrolled due to accessibility, the free services offered, or consultations with project physicians. Since the study population was not selected randomly, it might have varied, possibly resulting in a non-representative sample, which limits the generalization of the study findings. However, the results are consistent with those of previous studies, indicating a low degree of selection bias.

## Conclusions

This study revealed a high prevalence of malaria infections in the study villages, with significant variations in prevalence and risk observed at the district/regional and village levels. The odds of malaria infection were higher among males, children under 5 years, and schoolchildren. Individuals from households with low SES, lack of bednet use, and living in poorly constructed houses with open windows and holes in the walls also had higher odds of malaria infection. These findings highlight the need for strong initiatives to control malaria in these communities, with a particular focus on and targeting both symptomatic and asymptomatic infections. Efforts should also prioritize identifying malaria hotspots and vulnerable high-risk groups requiring more intensified control measures such as scaling up vector control strategies, including ITNs and other effective interventions to control and eliminate malaria.

## Supplementary Information

Additional file 1.

## Data Availability

Data supporting the main conclusions of this study are included in the manuscript.

## Abbreviations

ACT: Artemisinin-based combination therapy
AIC: Akaike information criterion
aOR: Adjusted odds ratio
ART-R: Artemisinin partial resistance
CI: Confidence interval
cOR: Crude odds ratio
CSS: Cross-sectional survey
DBS: Dried blood spots
GPS: Geographical positioning system
ICC: Inter-cluster correlation coefficient
IDs: Identification numbers
IPTp: Intermittent preventive treatment during pregnancy
IQR: Interquartile range
IRS: Indoor residual spraying
ITNs: Insecticide-treated nets
LSM: Larval source management
MRCC: Medical Research Coordinating Committee
MSMT: Molecular surveillance of malaria in Tanzania
NIMR: National Institute for Medical Research
NMCP: National Malaria Control Programme
ODK: Open Data Kit software
PCA: Principal component analysis
PO-RALG: President’s Office, Regional Administration and Local Government
RDTs: Rapid diagnostic tests
SES: Socio-economic status
SP: Sulfadoxine-pyrimethamine
WHO: World Health Organization
WHO-AFRO: WHO African Region
